# Canine root/cortical bone relation (CRCR) and the orthodontic tooth movement

**DOI:** 10.1038/s41598-022-14663-3

**Published:** 2022-06-23

**Authors:** Amr R. El-Beialy, Noha A. El-Ashmawi, Mohamed Abd El-Ghafour

**Affiliations:** Department of Orthodontics and Dentofacial Orthopedics, Faculty of Dentistry, Cairo University, Cairo, 11553 Egypt

**Keywords:** X-ray tomography, Cementum, Outcomes research

## Abstract

This observational study evaluates three-dimensionally the relation between the root of maxillary canine and overlying labial cortical plate of bone during orthodontic canine retraction. Eighty-four bilateral maxillary canines in 42 patients were retracted in the extraction space of first premolars, using conventional orthodontic NiTi retraction spring delivering 150gm. Three-dimensional evaluation at the cusp tip, root apex, and the overlying cortical bone was done based on Classification of Root/Cortical bone relation (CRCR) before and after canine retraction. 168 observations of the canines pre- and post-retraction showed a mean distal movement of the canine cusp tip of 3.78(± 2.05) mm, while the canine root apex was almost stationary. Scarcely, 5.4% of the canine roots and root apices persisted in the medullary bone during retraction, while 16.1% contacted the overlying cortical bone. Fenestration of the overlying cortical bone by the canine roots or root apices occurred in 78.6% of the sample. The unembellished intimacy between the canine root and apex to the overlying thick dense cortical bone might have the decelerating effect on the maxillary canine retraction. The natural bone plate labial to the maxillary canine root did not yield infront nor enlarge due to canine retraction, but else defeated the current orthodontic biomechanical implementation.

## Introduction

Orthodontic tooth movement (OTM) occurs as a consequence of force applied to the teeth, that results in a cellular response followed by modeling of the periodontal housing of the teeth, and hence teeth movement^1–3^. The response of the alveolar bone to the orthodontic tooth movement (OTM) proceeds uneventful provided that OTM is ensued within the safe boundaries of the alveolar housing. On the contrary, any violation of the alveolar bone boundaries due to an unduly OTM is faced with tenacious acellular cortical bone. This dense cortical bone does not yield nor enlarge to maintain a protection for the moving roots, with consequent deleterious effects on the periodontium^4–9^.

The mutual relation and support between the teeth and the alveolar bone showed that moving the teeth into the alveolar bone results in increased alveolar bone thickness^10–12^. On the other hand, moving the teeth outside the borders of the housing alveolar bone results in undesired sequlae^4,6,10,13–22^. These iatrogenic sequlae are the clinical manifestations of deterioration of the periodontium. These manifestations are expressed as gingival recession^23–26^, and dehiscence of the posterior labial cortical bone due to over-expansion of the maxillary posterior teeth^17,27–33^. The same pattern of disruption and dehiscence of the alveolar plate of bone labial to the incisors is indisputable following severe labial tipping of the lower incisors or uncontrolled tipping of the upper incisors^14,17–20^. In addition to the most deleterious effect; external root resorption^18,36,37^. Additionally, it was reported in an untreated sample that the lack of harmony between the alveolar bone thickness and the bucco-lingual dimension or the position of the teeth, is expressed as dehiscence, fenestration and potential gingival recession on the long term^6–9,16,29,34,35,38,39^. Although this evidence seems logical, it is alarming.

For decades, there has been an undocumented belief that the pathway of canine retraction occurs within the alveolar bone envelop parallel to the overlying cortical bone without any violation of the covering thick dense cortical bone. Accordingly, any attempt towards studying the effect of canine retraction on the overlying cortical bone might have sounded illogical. On the other hand, the severe distal tipping pattern of the canine during retraction has always been provoking^40–42^. These contradicting; belief and evidence, prompted the investigation of canines retracted using conventional mechanics, with particular emphasis upon the relation of the canine root and root apex to the overlying cortical bone in a three-dimensional nature. This relationship is introduced in a new classification titled; Classification of the Root/Cortical Bone Relation (CRCR). Hence, the aim of this observational study was to investigate and classify the relationship between the canine root and the labial cortical plate of bone during maxillary canine retraction using conventional orthodontic mechanics.

## Results

The intra-examiner and inter-examiner reliability values demonstrated excellent concordance, with weighted Cohen’s kappa coefficients between each 2 observers.

**Figure 1:**
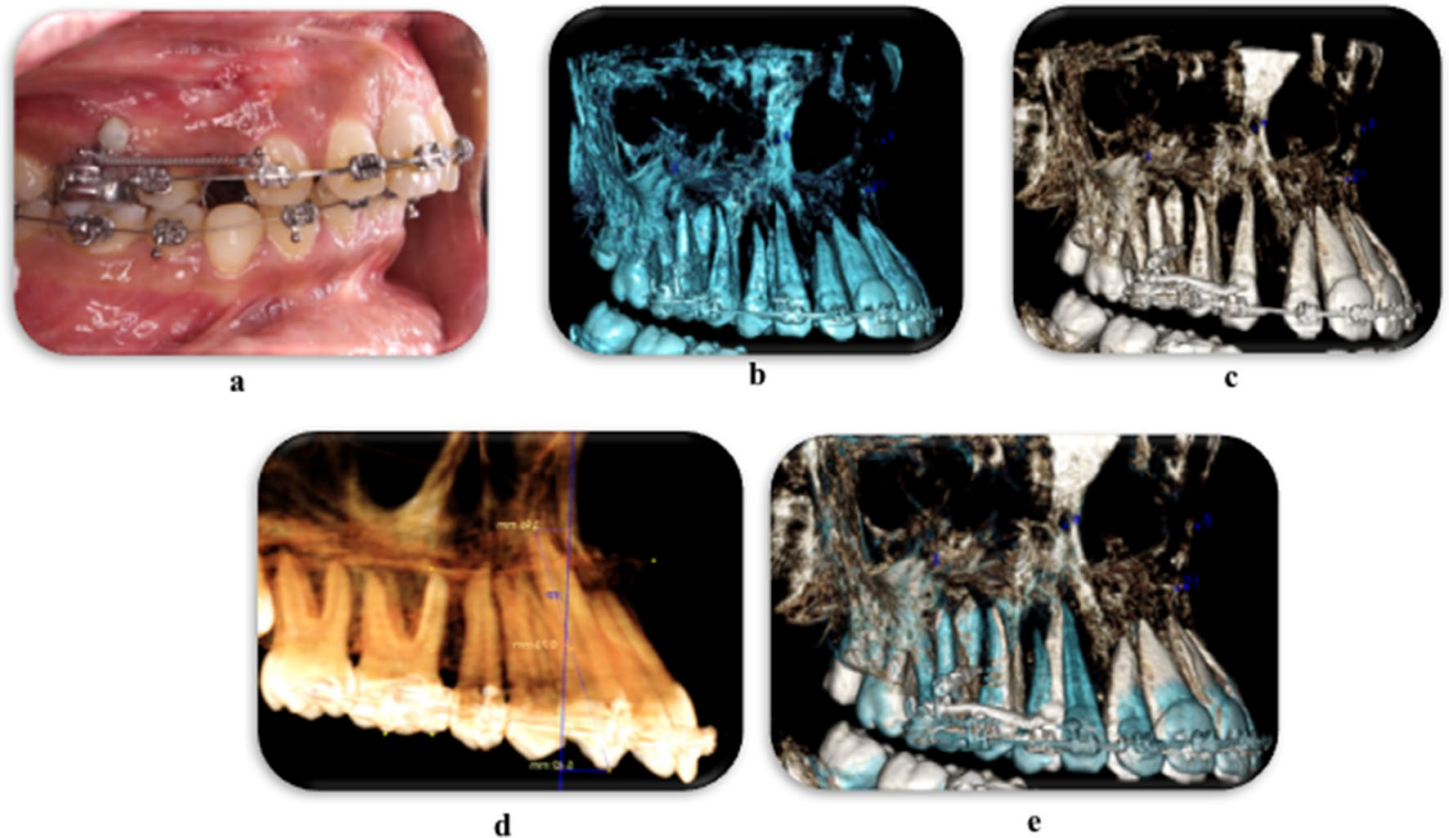
**(a)** Maxillary canine retraction using conventional mechanics, **(b)** maxillary arch CBCT before canine retraction, **(c)** maxillary arch CBCT after canine retraction, **(d)** distances traveled by canine cusp tip and root apex on CBCT, **(e)** superimposed CBCTs before, and after canine retraction showing the amount of canine tipping.

Bilateral maxillary canines, 84 in number, were retracted in the space of extracted first premolars using conventional mechanics (Fig. 1a). The total number of observations of the canine root and root apex in the pre and post retraction positions were 168 for 84 maxillary canines, with no drop-outs (Fig. 1b,c). This number was equally divided between the pre-retraction and post-retraction positions and evenly distributed among the right and left sides. The Classification of the Root/Cortical Bone Relation (CRCR) is summarized in Table 1.

**Table 1:**
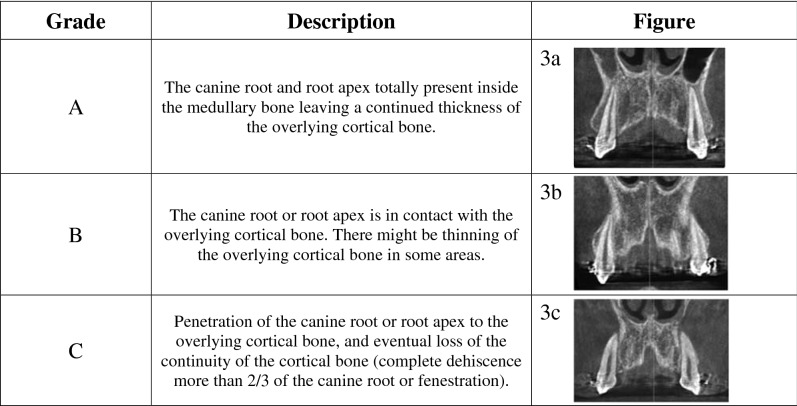
Classification of the root/cortical bone relation.

After 4 months of continuous canine retraction, the mean distal movement of the canine cusp tip was 3.78 mm (± 2.05) in a distal direction, while the mean movement at the root apex was 0.27 mm (± 1.29) in the mesial direction. The canine showed distal tipping angle of 8.46º ± 4.73º (Table 2, Fig. 1d,e).

**Table 2:** Distances travelled by the canine cusp tip and root apex and canine tipping after 4 months of active retraction.

Groups	Minimum	Maximum	Mean	Standard deviation
Mean movement of the canine cusp tip (mm)	0.94	8.69	3.78	2.05
Mean movement of the canine root apex (mm)	−3.53	3.21	−0.27	1.29
Mean tipping angle of canine (degree)	0.57	25.03	8.46	4.73

Positive sign means distal movement (mm), negative sign means mesial movement (mm).

**Figure 2:**
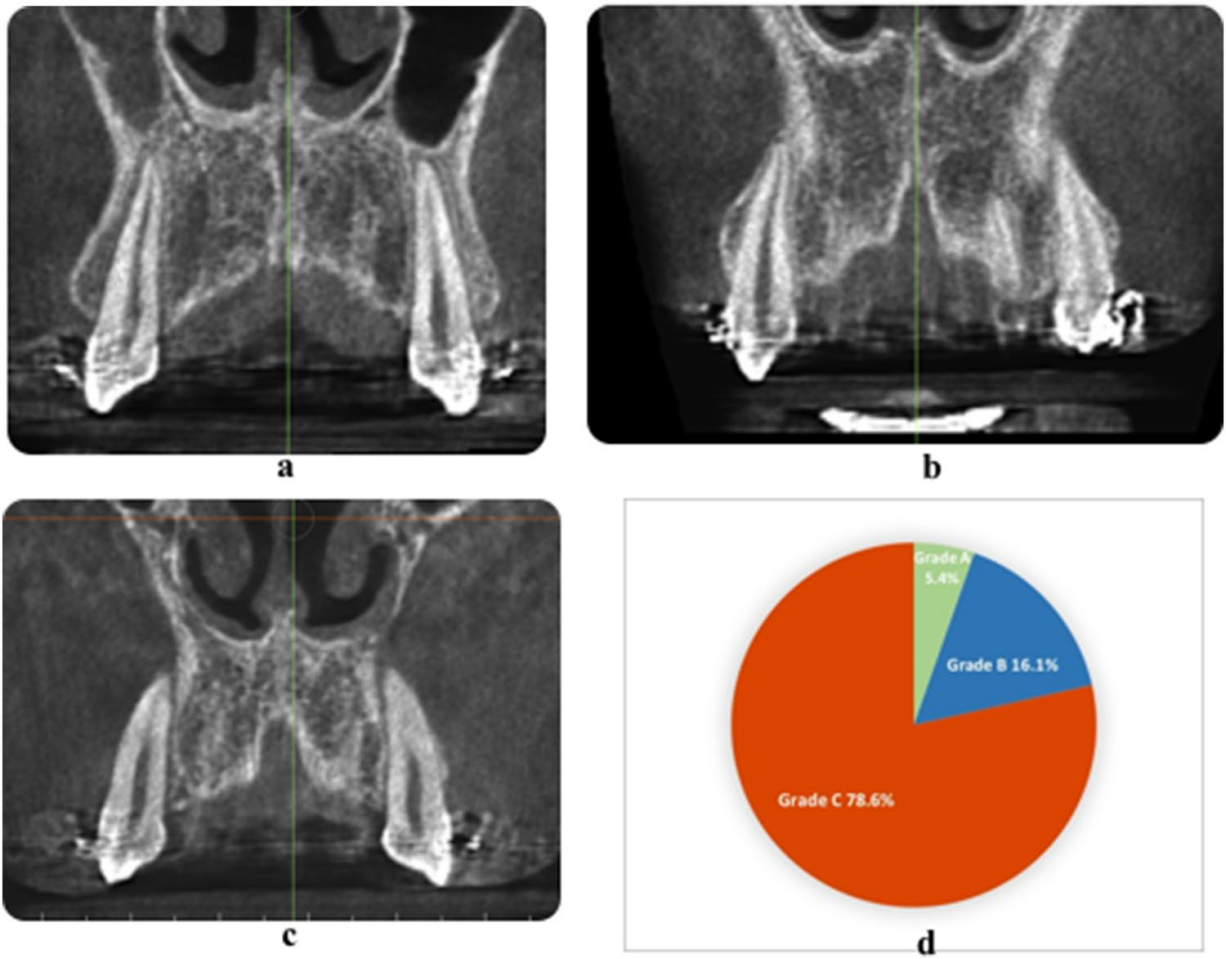
**(a)** CRCR Grade A: the canine root and root apex totally present inside the medullary bone leaving a continued thickness of the overlying cortical bone. **(b)** CRCR Grade B: the canine roots or root apices in contact with the overlying cortical bone, with thinning of the overlying cortical bone. **(c)** CRCR Grade C: penetration of the canine roots of the overlying cortical bone, and loss of the continuity of the cortical bone (fenestration or deep dehiscence). **(d)** Proportional percentage of each of CRCR grades.

**Figure 3:**
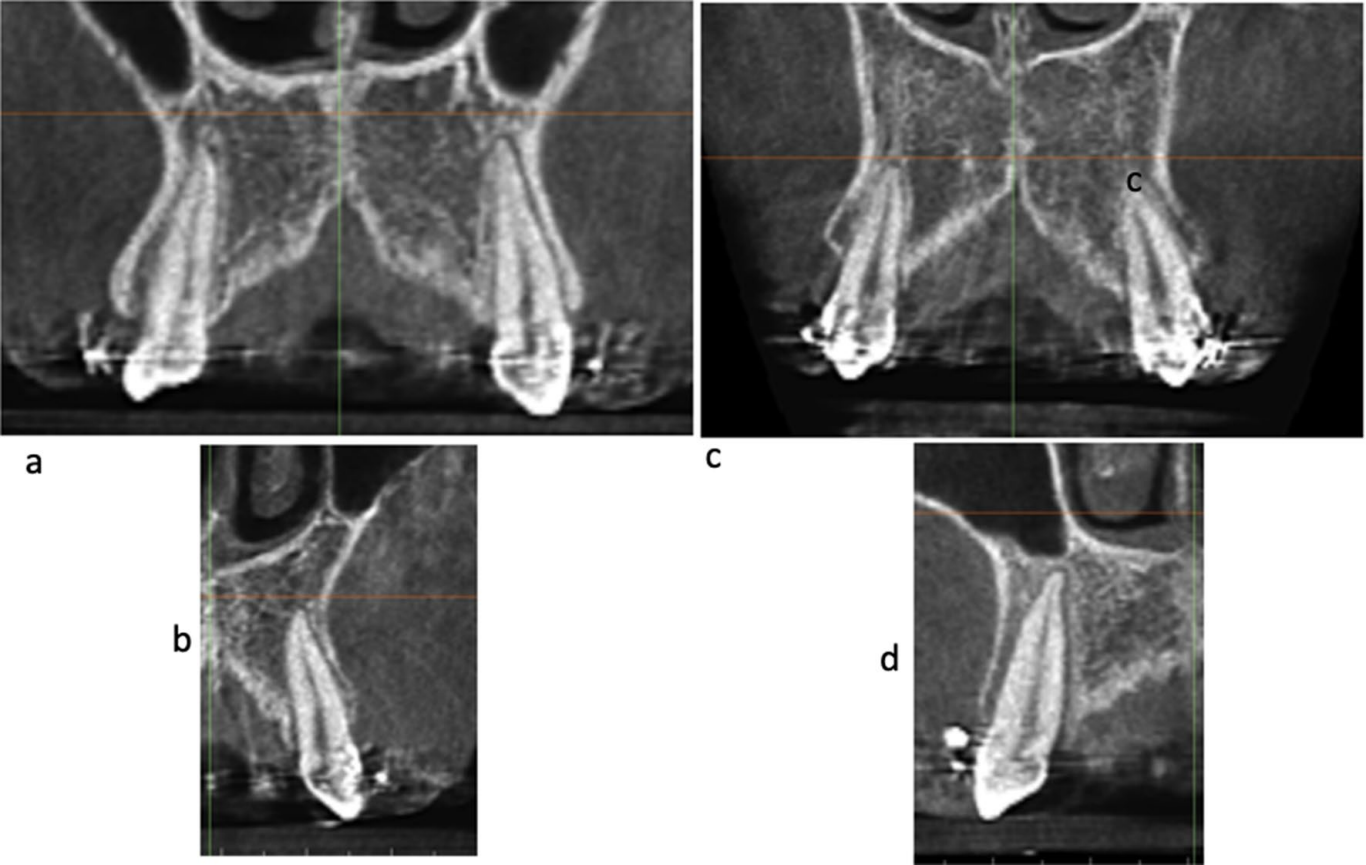
Examples of CRCR Grade A.

**Figure 4:**
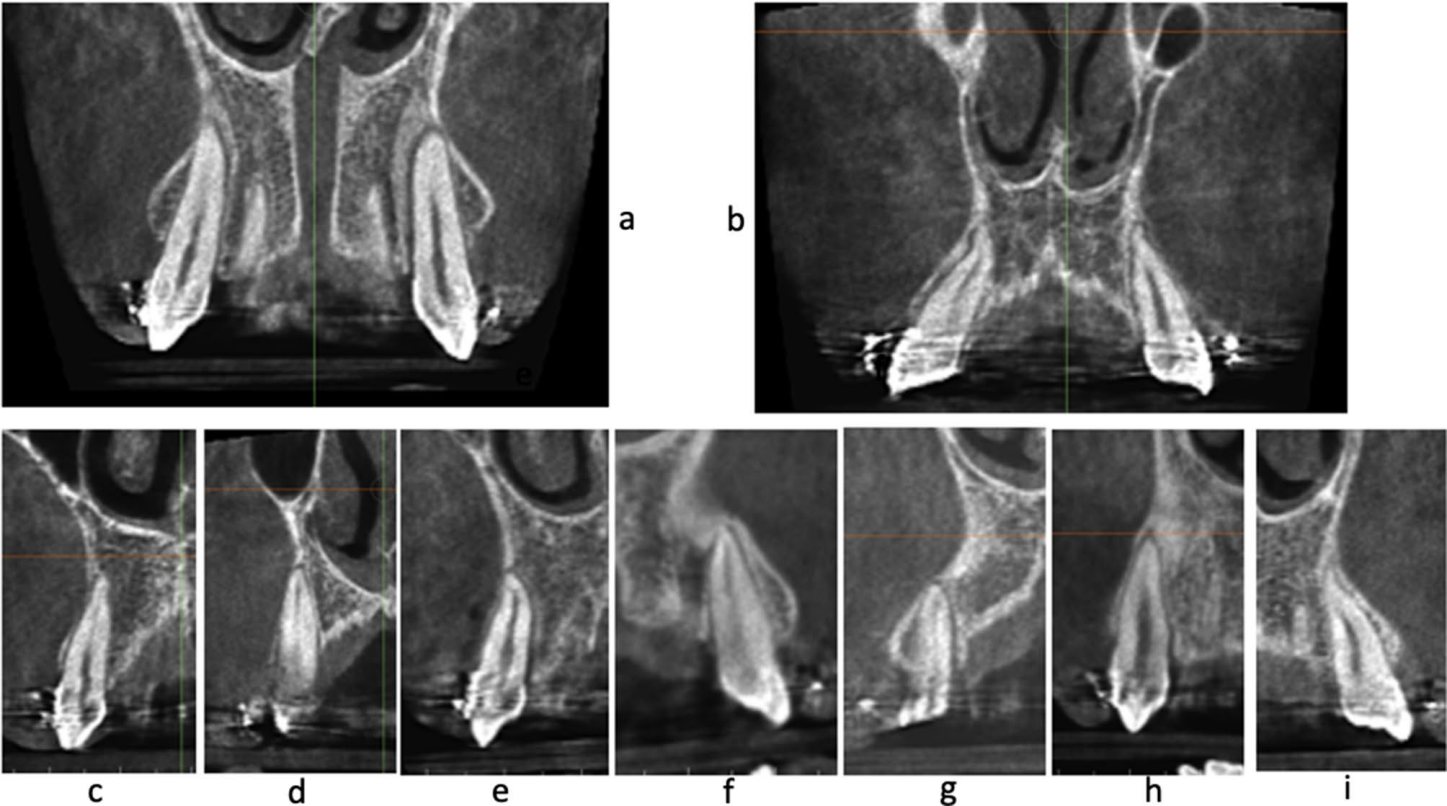
Examples of CRCR Grade B.

**Figure 5:**
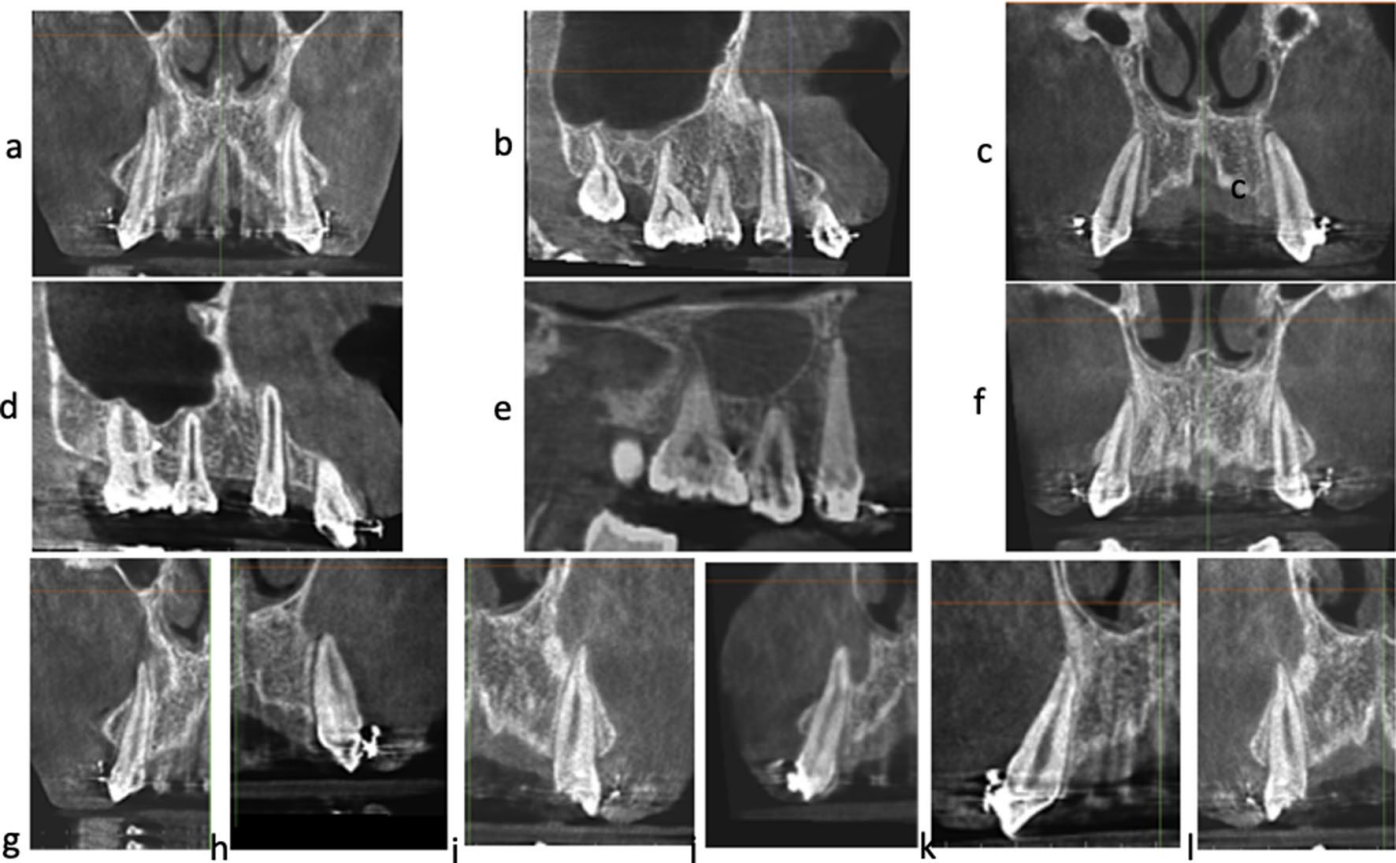
Examples of CRCR Grade C.

A simple and genuine classification of the relation between roots of the teeth and the covering cortical alveolar bone is introduced in this article, whereby the intimacy between the teeth roots and the overlying cortical bone plate is graded. According to the Classification of the Root/Cortical bone Relation (CRCR) aforementioned (Fig. 2a–c), the number of canine roots and apices belonging to each grade (Fig. 2d), in the pre-retraction and post-retraction observations showed the preponderance of grade C canines over grades A or B. Grade A included nine canine roots (5.4%) (Fig. 3a–d), while Grade B included 27 canine roots (16.1%) of the total canine observations (Fig. 4a–i). On the other hand, Grade C showed the highest preponderance of 132 canines which represent 78.6% of the total number of canine observations (Fig. 5a–l) (Table 3).

**Table 3:** Shows the total number of the canine root/cortical bone grades in the pre- and post-retraction CBCT’s.

	Pre-retraction	Post-retraction	Total	Total %	Percentages
Grade A	5	4	9	5.3	5.4
Grade B	19	8	27	16.1	94.6
Grade C	60	72	132	78.6	

**Figure 6:**
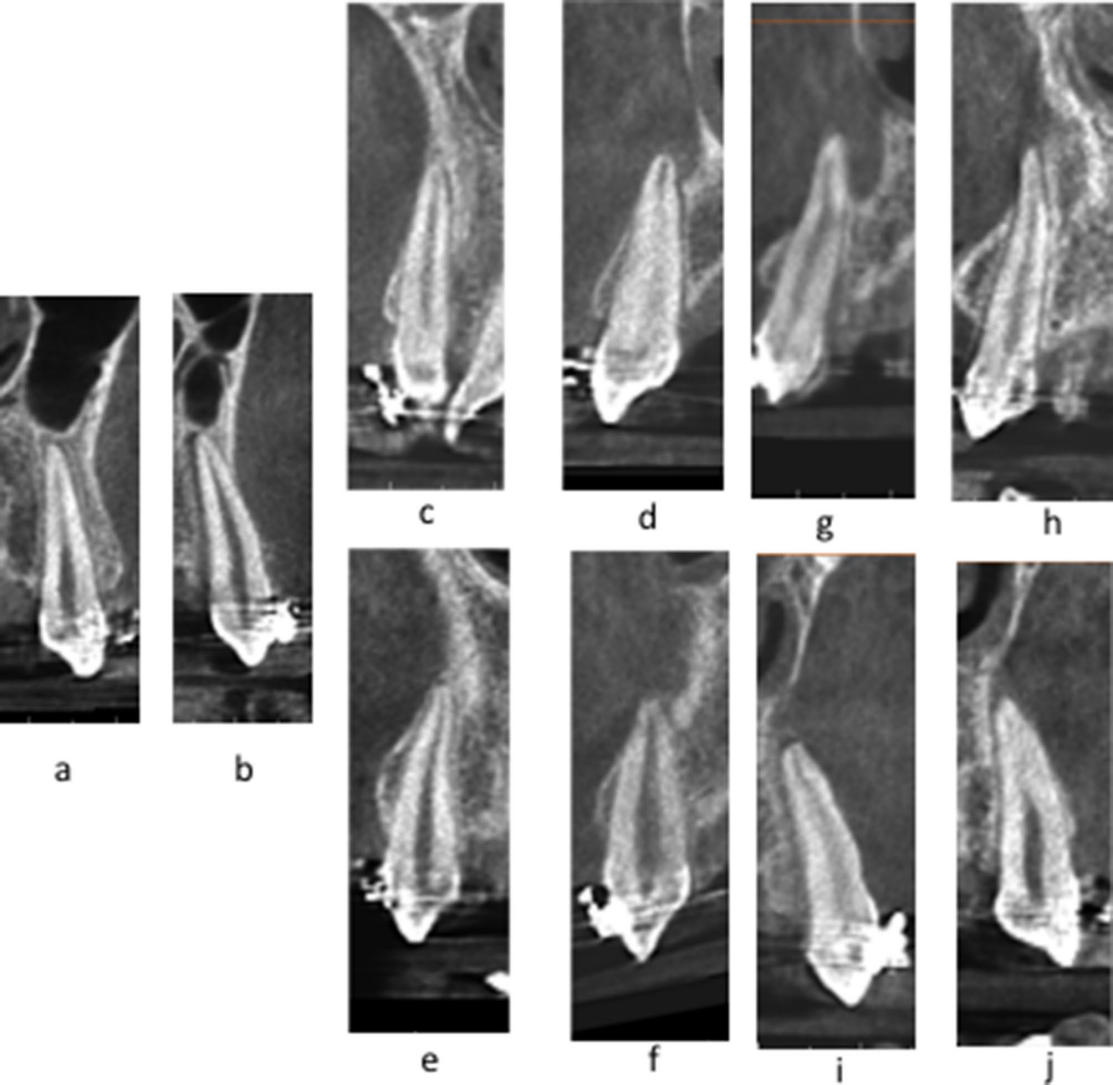
**(a,b)** Canines of the same patients in Grade A in the pre-retraction position **(a)** changed to grade C in the post-retraction position **(b), (c–f)** canines of the same patients in Grade B in the pre-retraction position **(c,e)** changed to grade C in the post-retraction position **(d,f)**, **(g–j)** canines of the same patients in Grade C in the pre-retraction position**(g,i)** remained as grade C in the post-retraction position **(h,j)**.

The change of the canine roots and apices from the pre-retraction grade to the post-retraction grade represents the following findings (Table 4). Four out of the five Grade A canines in the pre-retraction position has transformed to grade C in the post-retraction position (Fig. 6a,b). The same happened for 11 out of the 19 Grade B canines in the pre-retraction position which transformed to grade C in the post-retraction position (Fig. 6c–f). Almost all grade C canines retained their grade C in the post-retraction observation (Fig. 6g–j).

**Table 4:** Showing the change of the grade of the canine root and root apices from the pre- to the post- retraction position for the same subjects.

Pre	Post		
	Grade A	Grade B	Grade C
Grade A (5)	1	0	4
Grade B (19)	2	6	11
Grade C (60)	1	2	57

**Figure 7:**
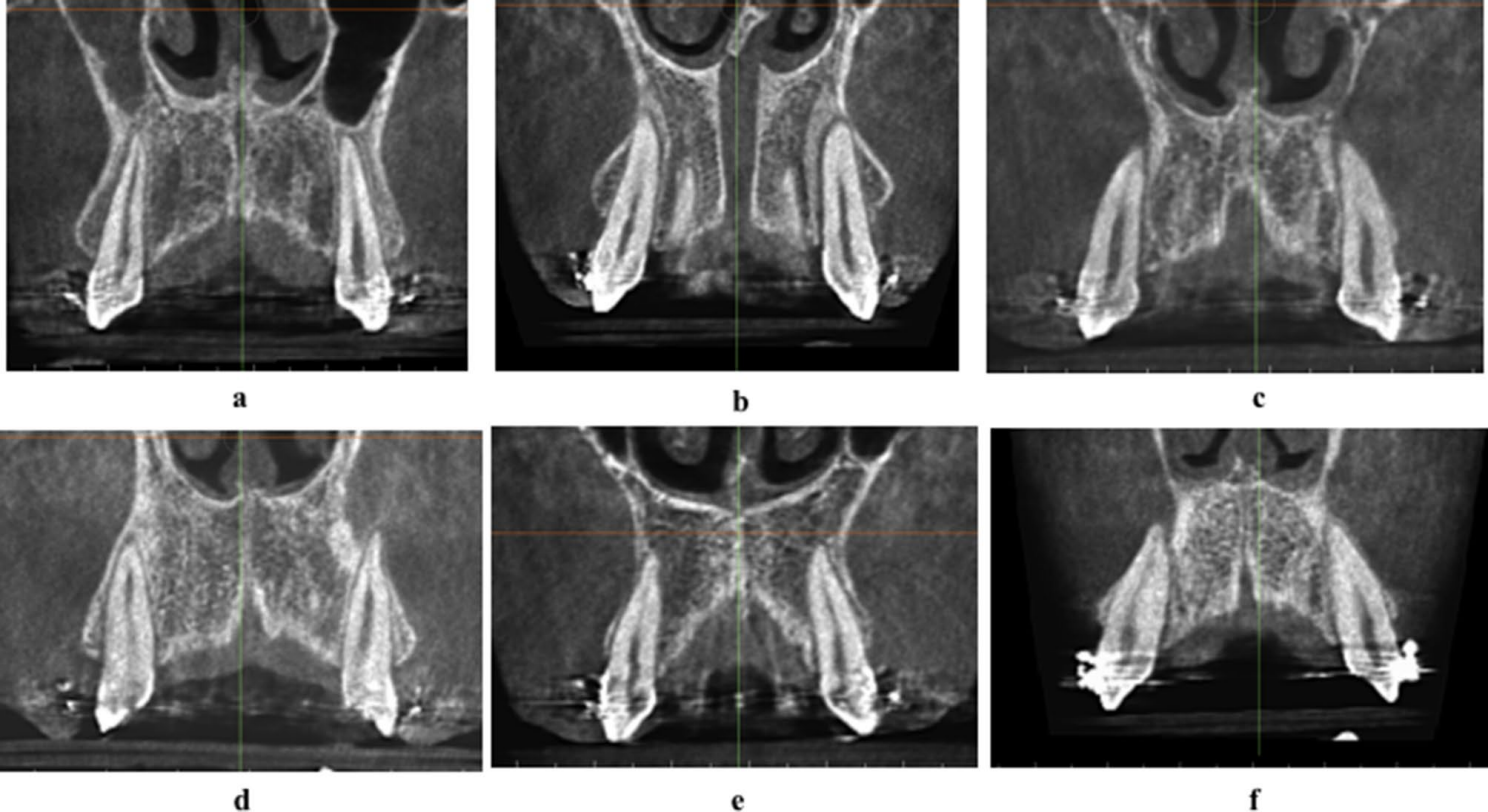
**(a)** Identical grade A of CRCR bilaterally. **(b)** Identical grade B of CRCR bilaterally. **(c)** Identical grade C of CRCR bilaterally. **(d)** Different grades of CRCR bilaterally (Right grade A/Left grade C). **(e)** Different grades of CRCR bilaterally (Right grade B/Left grade A). **(f)** Different grades of CRCR bilaterally (Right grade C/Left grade B).

In 58 arches out of the 84 inspected arches (pre-and post-retraction) of the patients, the same grade of the canine root/cortical bone relation occurred bilaterally (Fig. 7a–c), while different grades between the right and left sides occurred in 26 arches (Fig. 7d–f).

## Discussion

Controlling orthodontic tooth movement (OTM) and shortening the orthodontic treatment time is a valid demand amid the orthodontic community^43–45^. Although abundant attempts have been reported for acceleration of OTM, from altering the retraction wire^46–49^, to changing the bracket design^49,50^ and up to the pharmacological^51,52^, physical^53,54^ and surgical^55–59^ approaches, the mystery of the untamed rate of OTM is still unveiled.

Although the canine tooth has been taken as a model for investigating OTM in a substantial number of researches, the differential rate of retraction of contralateral canines, and the severe tipping of the crown compared to the root movement was a common finding. Besides, though the difference in retraction pattern between the sliding and frictional mechanics which have been widely stated in the literature, there is no sound explanation of these phenomena^57,60^. This recurring finding triggered the assumption that a discreet factor might be controlling OTM. Hence, the current observational study was conducted to investigate three dimensionally the relationship between the canine root and the labial cortical plate of bone before and after maxillary canine retraction using conventional mechanics.

In the current observational study, 42 patients with 84 bilateral maxillary canines were enrolled. The patients’ orthodontic treatment plan necessitated extraction of maxillary first premolars and retraction of maxillary canines. Conventional orthodontic mechanics were applied using NiTi retraction spring delivering a force of 150 g. Three-dimensional CBCT were collected prior to and after canine retraction yielding 168 observations of the canine. The pre-retraction and post-retraction CBCT were superimposed on stable skeletal structures.

Similar to other studies^57,60^, results of the current study revealed that all the investigated 84 canines moved by severe crown tipping after 4 months of active canine retraction. Remarkably, the root apex of the canines showed a negligible amount of movement (Fig. 1e, Table 2).

**Figure 8:**
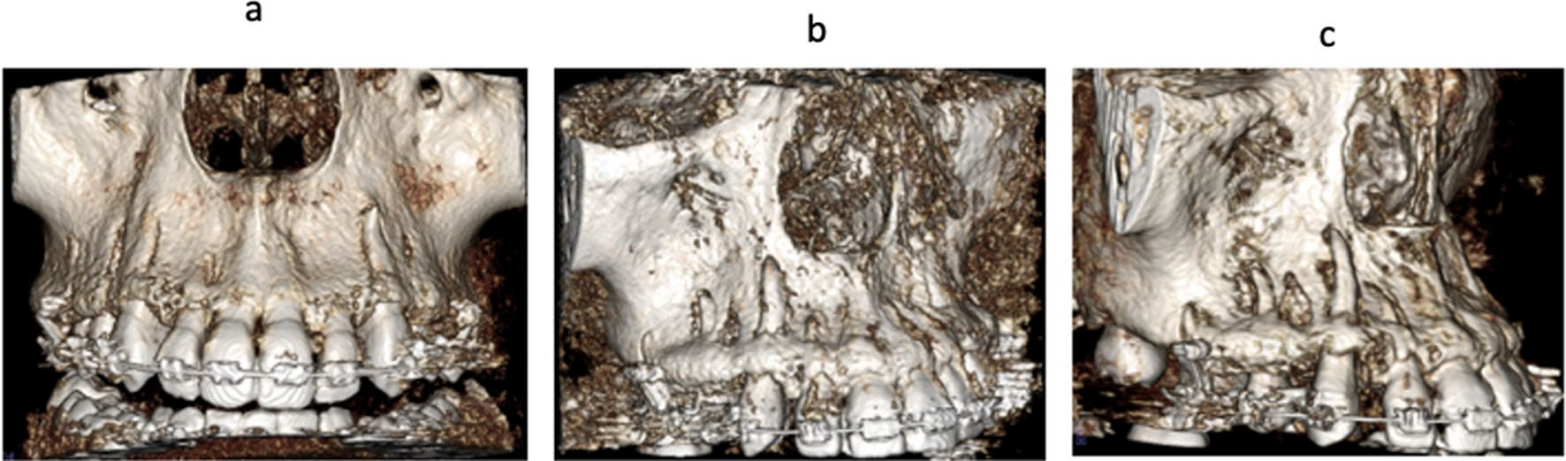
CBCT volumes examined in the 3D solid bone view.

When the CBCT volumes were examined in the 3D view, an outstanding relation between the canine apices and the overlying cortical bone was observed (Fig. 8a–c). Many of the canine apices and apical thirds pierced the overlying cortical bone, resulting in unambiguous dehiscence and fenestration. Since the 3D volumes are not accurate representation of the true reality^61^, the orthogonal sections were assessed. The orthogonal sections depicted distinctly and unmistakably the same findings of the 3D views (Figs. 2, 3, 4 and 5). There was an intimate relationship between the investigated canine root and apex to the overlying cortical bone (root/cortical bone relation). Accordingly, a classification for grading the relation between the canine root and apex with the overlying cortical bone was established (Table 1).

In this article a new and simple classification of the relation between roots of the teeth and the covering cortical alveolar bone is introduced. This Classification of Root/Cortical bone Relation (CRCR) comprised three grades, that were used to rate the pre-retraction and post-retraction canine root positions (Fig. 2a–c). Implementing the Classification of Root/Cortical bone Relation (CRCR; A, B & C grading) demonstrated in Fig. 2a–c, showed a clear discrepancy between grade C versus grades A or B. The investigated canine roots in grade C (78.6%) were more dominant than the other two grades. Unexpectedly, the percentage of canine roots and apices that belong to category A and B represented only 5.4% and 16.1% of the total sample respectively (Fig. 2d). Analyzing this data from a clinical point of view, almost 95% (Grades B + C collectively) of the total canine roots and apices had intimate relation to the overlying cortical bone (Table 3).

The anatomical nature of the cortical bone, being a thick dense cortical lamellar nature with minimal vascularity and reduced remodeling capabilities^62^ is its own advocate as a mean to halt the movement of the canine root apex. This nature of the cortical bone was implemented in the Tweed treatment philosophy that used to torque the dental roots towards the overlying cortical bone as a mean of anchorage reinforcement^5^. The findings of slow OTM, severe tipping of the canines, negligible movement of the canine roots and root apices together with the relation of the canine to the overlying dense avascular minimally remodeling cortical bone is interesting.

Curiously, comparing the pre-retraction to the post-retraction data for the same patients (Table 4), there is a pattern of deterioration of the grade of the Root/Cortical bone Relation towards a situation of more engagement to the overlying cortical bone. As shown in Table 4, out of the five roots and apices that belong to category A in the pre-retraction position, four of these same canines deteriorated into category C in the post-retraction situation (Fig. 6a,b). And 11 out of the 19 grade B canines in the pre-retraction position deteriorated to category C post-retraction (Fig. 6c–f). Almost all the canine roots and apices which started in category C continued in category C (Fig. 6g–j).

A biomechanical justification to this phenomenon has more than one aspect. The nature of the canine being at the corner of the dental arch makes the canine root vulnerable to bony fenestration upon retraction. The lack of synchronization between the anatomy of the canine root and the alveolar bone thickness in the malocclusion sample is another reason. Buccal root torque expression due to engaging a rectangular arch wire in a rectangular bracket slot might be a contributor^63^. Besides, the play between the arch wire and the bracket slot might have permitted some lingual crown tipping of the canine with subsequent buccal root torquing, and hence more engagement of the canine root into the buccal cortical bone.

We believe that the presence of any relation of the canine root or apex to the overlying cortical bone in either the pre- or post-retraction time point would have a braking influence on the journey of canine root. The pattern of the deterioration proves there is more engagement of the canine root into the overlying buccal cortical bone during retraction. The existence of a cortical penetration in the post-retraction position, indisputably meant that the canine root has passed through the phases of contact followed by embracement in the overlying alveolar bone before reaching this position of cortical piercing and fenestration. The impeding effect of this phenomenon on the canine movement cannot be over-stated.

In the current study, the grading was measured between the cortical bone and the canine root and apex excluding the coronal third of the root because the coronal third of the canine teeth was proved to be denuded of bone coverage in untreated orthodontic patients^39^.

An intimidating awareness is the absence of any clinical sign during the retraction of the canine that could have implied the presence of any fenestration or cortical bone penetration. No bulging nor deterioration of the periodontal condition nor fenestration was ever clinically pulpable during the course of this study.

As far as our literature search, this study is the first attempt at investigating and grading the relation of canine root and apex to the labial cortical bone plate. It proposes a simple qualitative grading system named, Classification of Root/Cortical Bone Relation (CRCR). Our proposed assumption is as follows; since the canine root apex depicted negligible movement, and simultaneously, almost all the sample showed intimate relation between the canine root and apex with the overlying alveolar bone, then the CRCR might have a considerable influence on the rate of canine retraction. Considering the relation of the roots to the overlying cortical bone throughout planning the orthodontic treatment mechanics might open the door for a different philosophy for OTM.

The current article describes a phenomenon that leaves more questions than answers. Does this peculiar relation between the canine root and root apex with the overlying cortical bone exist in every case involving canine retraction? Does this discreet factor in the background manipulate OTM? Can we blame this relation for the decelerated OTM and the distal crown tipping accompanying canine retraction? Do we expect this incipient phenomenon to take place in the background in routine orthodontic cases in the absence of any clinical sign? Should the belief that OTM is taking place as assumed in the medullary bone be reconsidered, or is an ectopic journey taking place? Is there something erroneous in using the canine retraction research model in studies assessing acceleration of OTM? Based on the current findings, we believe that Orthodontic tooth movement is worth a 3D revisit. Additional studies are required to further investigate the current phenomenon.

## Methods

The research protocol of the current study was revised and approved by the Research Ethics Committee, Faculty of Dentistry, Cairo University in Egypt. All methods were performed in accordance with the relevant guidelines and regulations. Informed consent was signed by all participants prior to the initiation of this study.

### Study sample

The sample consisted of 42 patients with a mean age of 20 (± 3.85). Eighty four maxillary canines were investigated. The inclusion criteria were bimaxillary dentoalveolar protrusion, or class II division 1 malocclusions (Table 1) which necessitated extraction of upper first premolars and canine retraction, besides, healthy periodontium and normal medical condition.

Orthodontic treatment started using fixed orthodontic straight wire brackets, 22 × 28 mil slot, Roth prescription (Ormco-Mini 2000 brackets) with zero-torqued canine brackets. To ensure maximum retraction, indirect anchorage was secured with mini-screws. Leveling and alignment was done with an arch wire sequence tailored to each case until an arch wire 16 × 22 mil stainless steel is passively ligated to the maxillary arch brackets. At this stage, extraction of bilateral maxillary 1st premolars was done, and canine retraction was initiated on the same day using NiTi coil spring delivering a force of 150 g^40,46–49,55–57,64^ (Fig. 1a).

### Variables measured

In order to assess the movement of the canine three dimensionally, Cone Beam Computed Tomography (CBCT-(Cranex^®^ 3D–3D dental imaging system—Soredex with the following parameters: Resolution (voxel size): 0.3\0.3 mm, exposure time: 4860 ms, anode voltage: 89.8 kV, field of view (FOV): 8 × 8 cm, anode current: 10 mA and sensor: CCD-detector), restricted to the maxillary arch was done immediately before (Fig. 1b) and after (Fig. 1c) canine retraction. CBCT analysis was performed using Invivo5 (Anatomage Inc. San Jose, CA 95110) application. The distances traveled by the canine cusp tip and root apex were measured on the pre- and post-retraction CBCT (Fig. 1d) with reference to 3D planes (midsagittal plane at ANS, PNS and incisive foramen, and the frontal plane perpendicular to that plane at the incisive foramen) and superimposition of the pre- and the post-CBCT was done (Fig. 1e).

The canines were examined on the CBCT volumes in the 3D solid bone view (Fig. 8a–c), followed by evaluation of the orthogonal sections. The relation between the canine root and root apex to the overlying cortical bone; classification of the root/cortical bone relation; CRCR, was established into three grades (Table 2).

Calibration of the assessors’ rating was done using random anonymous 40 images, in which 3 orthodontists assessors (A.B., N.A., M.A.) openly classified the canine grade with the overlying cortical bone on the orthogonal slices, and any queries regarding the different grading stages were discussed, until a consensus of definition was reached. After this calibration of the assessors, each image of the study, was graded anonymously by the three assessors sitting together under the same working conditions.

### Statistical analysis

For every patient, the change of the CRCR grade from the pre-retraction to the post-retraction positions was computed. The mean movement of the canine cusp tip and root apex and the amount of tipping were calculated. To test the concordance between the observers, Cohen’s kappa coefficient measures of agreement was performed.

## Conclusion

Within the limitation of the current observational study the following can be concluded:

1. Classification of the Root/Cortical Bone Relation (CRCR) is a valid diagnostic tool to assess the relation between the root surface and the adjacent cortical bone.
2. Most of the observed canines had a grade C in the CRCR classification denoting a bony fenestration of the canine root and the overlying cortical bone.
3. After maxillary canine retraction, worsening of the CRCR scores had occurred in most of the observed canines and resulted in more contact between the root surface and the cortical bone. This might be a reason of canine tipping and retardation of OTM.

### Clinical implications

The dehiscence reported, might give some explanations for the current pace and pattern of the canine OTM and the accompanied consequences on the associated alveolar bone. New modalities for retraction of the maxillary canines should be investigated, to preserve the canine root inside the medullary alveolar proper, which might be an initial step towards accelerating the OTM.

## Data Availability

Raw data were generated at Faculty of Dentistry, Cairo University. Derived data supporting the findings of this study are available from the corresponding author upon request.
